# Editorial: Molecular and multi-omic approaches in understanding cancer biology and anticancer therapies: current perspectives and new challenges

**DOI:** 10.3389/fphar.2023.1236158

**Published:** 2023-06-15

**Authors:** Basel A. Abdel-Wahab, Yosra A. Helmy, Essa M. Saied

**Affiliations:** ^1^ Department of Medical Pharmacology, Faculty of Medicine, Assiut University, Assiut, Egypt; ^2^ Department of Veterinary Science, College of Agriculture, Food, and Environment, University of Kentucky, Lexington, KY, United States; ^3^ Institute for Chemistry, Humboldt Universität zu Berlin, Berlin, Germany

**Keywords:** multiomics, colon cancer, prostate cancer, ovarian cancer, breast cancer, lung cancer

## 1 The aim and scope of this Research Topic

A holistic understanding of cancer biology and pathophysiological features is crucial. Multi-omics approaches integrate multiple datasets to understand cancer molecular and clinical features. This data-driven study reveals the complexity of cells and their environment, improving survival prediction and therapeutic outcomes. This specific Research Topic is to encourage talented researchers working in the field of multi-omics and cancer to publish their work with Frontiers in Pharmacology. The Research Topic features 11 articles, including 10 original and one review articles, in a multidisciplinary collaboration among multiomic-bioinformatic, *invitro*, and clinical studies. These articles cover several cancer types, including pancreatic, ovarian, cervical, colorectal and lung cancer.

## 2 Overview of contributors

Necroptosis is a new target for cancer immunotherapy, enhancing tumor immunogenicity. Fang et al. through a comprehensive analytical study determined necroptosis subtypes and investigate the roles of necroptosis in pancreatic cancer therapy. Immunological cell infiltrations, immunological checkpoints, HLA molecules, and the cancer-immunity cycle were used to assess the immunogenic properties. Authors identified five subtypes of necroptosis in pancreatic cancer, with diverse prognosis, immunogenicity, and chemosensitivity. Future research should evaluate its relevance in combined therapeutic regimens and chemotherapy choices.

Ovarian cancer (OC) treatment still needs more molecular biomarkers for accurate prognostic and therapeutic decision-making. Assidi investigated the expression patterns of N-cadherin (N-CAD), which has been demonstrated to be overexpressed in numerous advanced carcinomas and determined their links with the clinicopathological characteristics of OC patients and assessed its prognostic value. N-CAD overexpression in OC is associated with a poor prognosis, as evidenced by increased recurrence and mortality rates, in addition to its molecular role to distant metastasis Assidi.

Cervical cancer, is the fourth most common cancer globally. In contrast to conventional interventions, Kori et al. focused on inflammatory proteins that classify cervical cancer patients by considering individual differences between cancer patients. Also, authors repurposed anti-inflammatory drugs using gene signature reversal and molecular docking. They suggested 5 novel drugs (aldosterone, BMS-345541, etodolac, hydrocortisone, and prednisone) for the treatment of both HPV subtypes, as well as 4 novel anti-inflammatory drugs (AS-601245, betamethasone, narciclasin, and methylprednisolone) for the treatment of HPV-16.

Endometriosis is a chronic, estrogen-dependent, inflammatory disease with an unknown etiology. characterized by the outward development of endometrial tissue. Integrated bioinformatics analysis was implemented by Wang Z. et al. to disclose the underlying molecular mechanisms of this disease. reveals the underlying molecular mechanisms of this disease. They identified four endometriosis-specific genes, predicting 51 potential drugs and revealing immunological function associations. Endometriosis has been classified into three subtypes with distinct mechanisms and immune characteristics. This study identified the characteristic genes and new molecular subtypes of endometriosis, thereby contributing to its early diagnosis and treatment.

Lung adenocarcinoma (LUAD) is the most prevalent histological subtype of lung cancer, and regulatory cell death is an attractive target for cancer therapy. Recent research suggests that cuproptosis being a promising target for cancer therapy. Nonetheless, the function of cuproptosis-related genes (CRGs) in the LUAD process remains unknown. Wang S. et al. found that DLD, LIAS, PDHB, DLAT, and LIPA1 were central genes in 10 differentially expressed CRGs enriched in mitochondrial respiration-dependent cell death, providing insights for treatment and immunotherapy drugs targeting cuproptosis.

A member of the transcriptional enhancer factor (TEF) family of transcription factors, TEA domain transcription factor 4 (TEAD4) is linked to the development and progression of several malignancies, including lung adenocarcinoma (LUAD). Nonetheless, the role of this gene in the progression of LUAD unclear. Gong et al. via gene analysis found that TEAD4 was substantially correlated with LUAD patients’ poor prognosis. Moreover, there was a robust correlation between high TEAD4 expression and immunotherapeutic resistance. This study revealed that TEAD4 is a predictor of prognosis related to immune regulation and a novel therapeutic target for LUAD.

A very diverse malignant carcinoma is gastric cancer (GC). Lin et al. used exosome-based classification to help tailor treatment for gastric cancer (GC). They created an exosome-based gene signature and assessed immunological characteristics, immune checkpoint inhibitor responses, and genetic changes utilizing computational techniques. There were two clusters of exosome-relevant phenotypes (A and B), and phenotype B had a worse prognosis and an inflammatory tumor microenvironment (TME) than phenotype A. The exosome-based gene signature predicted GC prognosis and genomic changes. This research provides a conceptual framework to better understand the functions of exosomes in immune escape mechanisms and GC genomic changes.

Ferroptosis, an iron-dependent necrosis in cancer, is associated with poor prognosis, inflammatory tumor microenvironment, and susceptibility to chemotherapeutic drugs. A systematic analysis by Xiao et al. identified subtypes and highlights the potential for tailored care. The PCA computational approach was used to create the ferroptosis index. It was found that there were strong correlations between clinicopathological features and FPI. High FPI was also associated with a poor prognosis, an inflammatory tumor microenvironment (TME), and high susceptibility to chemotherapeutic drugs.

A growing body of research has shown the biological significance of oxidative stress in the tumorigenicity and progression of colorectal cancer (CRC). In their study, Cao et al. developed an oxidative stress-related signature to predict clinical outcomes and therapeutic responses in colorectal cancer patients. The signature was associated with defined genes as ACOX1, CPT2, and UCN, and showed potential for survival prediction. This could improve prognosis and adjuvant therapy decisions. Cao et al. work created an oxidative stress-related signature that can potentially improve prognosis prediction and adjuvant therapy decisions.

Prostate cancer (PRAD) is a deadly disease with drug resistance and poor prognosis. Li et al. studied somatic mutations, somatic copy-number changes (SCNAs), DNA methylation, and mRNA expression in multi-omics profiling for G protein-coupled receptors (GPCRs) in the primary PRAD patients, identifying four potential medicines and novel biomarkers for treatment. These findings from the multi-omics analysis of GPCRs offer fresh perspectives on the underlying mechanisms of primary PRAD and the potential of GPCRs in the creation of PRAD-specific treatment approaches.

Tumor resistance to therapy remains a significant barrier, primarily due to intratumoral heterogeneity. Single cell profiling tools can identify clones with similar characteristics, potentially improving long-term therapeutic response in brain tumors. Murdaugh and Anastas in their review investigated the potential for single cell multi-omic analyses to reveal mechanisms of glioma resistance to therapy and discussed opportunities to apply these methods to enhance long-term therapeutic response in pediatric high-grade glioma with few treatment options.

## 3 Conclusion

In conclusion, this Research Topic has provided original research and updated reviews of early-stage researchers related to multi-omics research in cancer etiology, and therapy. Our knowledge of the pathophysiology of many types of cancers and new treatment options are both furthered by these investigations. The evidence collected from this Research Topic is also expected to be translated into more precise and practical clinical approaches to predict and treat relevant human disorders in the future.

